# Comparison of four different intraosseous access devices during simulated pediatric resuscitation. A randomized crossover manikin trial

**DOI:** 10.1007/s00431-017-2922-z

**Published:** 2017-05-12

**Authors:** Karol Bielski, Lukasz Szarpak, Jacek Smereka, Jerzy R. Ladny, Steve Leung, Kurt Ruetzler

**Affiliations:** 1 MEDITRANS The Voivodship Emergency Medical Service and Sanitary Transport, Warsaw, Poland; 2 Department of Emergency Medicine, Medical University of Warsaw, Lindleya 4 Street, 02-005 Warsaw, Poland; 3 Department of Emergency Medical Service, Wroclaw Medical University, Wroclaw, Poland; 4 Department of Emergency Medicine and Disaster, Medical University Bialystok, Bialystok, Poland; 5 Department of Outcomes Research, Cleveland Clinic, Cleveland, OH USA; 6 Department of General Anesthesiology, Cleveland Clinic, Cleveland, OH USA

**Keywords:** Intraosseous access, Pediatric, Paramedic, Cardiopulmonary resuscitation, Simulation

## Abstract

The aim of the study was to compare the success rate, procedure time, and user satisfaction of pediatric NIO™ compared to Pediatric BIG®, EZ-IO®, and Jamshidi intraosseous access devices. This was a randomized, crossover manikin trial with 87 paramedics. The correct location of intraosseous access when using NIO, BIG, EZ-IO, and Jamshidi was varied and was respectively 100, 90, 90, and 90%. The time required to obtain intravascular access (time T1) in the case of NIO, BIG, EZ-IO, and Jamshidi was varied and amounted to 9 s [IQR, 8–12] for NIO, 12 s [IQR, 9–16] for BIG, 13.5 s [IQR, 11–17] for the EZ-IO, and 15 s [IQR, 13–19] for Jamshidi. The paramedics evaluated each device on the subjective ease with which they performed the procedures. The intraosseous device, which proved the easiest to use was NIO, which in the case of CPR received a median rating of 1.5 (IQR, 0.5–1.5) points.

*Conclusion*: Our study found that NIO® is superior to BIG®, EZ-IO®, and Jamshidi. NIO® achieved the highest first attempt success rate. NIO® also required the least time to insert and easiest to operate even by novice users. Further study is needed to test our findings in cadavers or human subjects. Based on our findings, NIO® is a promising intraosseous device for use in pediatric resuscitation.

**Table:** 

**What is Known:**
• *Venous access in acutely ill pediatric patients, such as those undergoing cardiopulmonary resuscitation, is needed for prompt administration of drugs and fluids*.
• *Intraosseous access is recommended by American Heart Association and European Resuscitation council if vascular access is not readily obtainable to prevent delay in treatment*.
**What is New:**
• *This simulated pediatric resuscitation compared performance of four commercially available pediatric intraosseous devices in a manikin model*.
• *NIO® outperformed BIG®, EZ-IO®, and Jamshidi in first attempt success rates and time of procedure among novice users*.

## Introduction

Vascular access is a vital component of pediatric emergency medicine, and peripheral access in children in general is more difficult than in adults [2, 10, 14]. Certain situations such as cardiac arrest and shock may render peripheral access impossible due to peripheral vasoconstriction. This is especially important, as any delay in establishing a venous access may delay medical interventions and potentially compromises patient outcomes [1].

Intraosseous access is a widely accepted approach to gain venous access during cardiopulmonary resuscitation (CPR) of a critically ill child. The American Heart Association (AHA) and European Resuscitation Council (ERC) advocate the use of intraosseous access during CPR if peripheral vein access is not immediately possible to achieve [3, 12]. The medullary spaces serve as a “non-collapsible vein” that allows safe, effective, and rapid administration of medications, fluids, and blood products [3, 4, 7, 11, 13]. First attempt success rates of a variety of devices, such as Pediatric BIG®, EZ-IO®, and Jamshidi, are reported to range between 55 and 97% [3].

In 2016, the Food and Drug Administration approved an IO device, NIO Pediatric (NIO-P; Persys Medical, Houston, TX, USA) for pediatric patients between 3 and 12 years old (https://www.accessdata.fda.gov/cdrh_docs/pdf16/K160805.pdf). The NIO-P is a pediatric version of NIO-Adult, a widely used intraosseous device for adults in both laboratory and clinical settings [22] (https://ps-med.com/clinical/). NIO-P is a sterile, single use device in a simple packaging that is ready for quick deployment. It comprises of an 18-guage needle that inserts into the tibia by a spring-loaded mechanism. Previous favorable experience with the adult NIO led us to evaluate the performance of the pediatric NIO-P [22].

There are limited data in head-to-head comparisons between the NIO-P and widely used Pediatric BIG®, EZ-IO®, and Jamshidi in the pediatric population, especially during ongoing CPR. Therefore, our aim was to compare the success rate, procedure time, and user satisfaction of NIO-P compared to pediatric BIG, EZ-IO, and Jamshidi intraosseous devices.

## Methods

### Study design

This study was designed as a randomized, crossover manikin trial. After obtaining study approval by the Institutional Review Board of the Polish Society of Disaster Medicine (approval no.: 23.11.2016.37), we recruited 87 paramedics with less than 1-year experience in Emergency Medical Service (EMS). The paramedics had not been trained on any of the intraosseous access devices before the study began. All paramedics signed an informed consent and the study was performed in January 2017.

### Simulation of the scenario

Each paramedic performed intraosseous access on a SimJunior advanced life simulator (Laerdal, Stavanger, Norway), which represents a model of a 6-year old boy. Simulators were equipped with a pediatric intraosseous leg (Laerdal, Stavanger, Norway). Subjects performed intraosseous access during simulated ongoing CPR. To standardize the difficulties resulting from the chest compressions, the mechanical chest compression device Lucas 3 (Physio-Control, Redmond, WA, USA) was used [23]. For each intraosseous attempt, the manikin was placed on the floor in a bright room.

### Devices

**Fig. 1:**
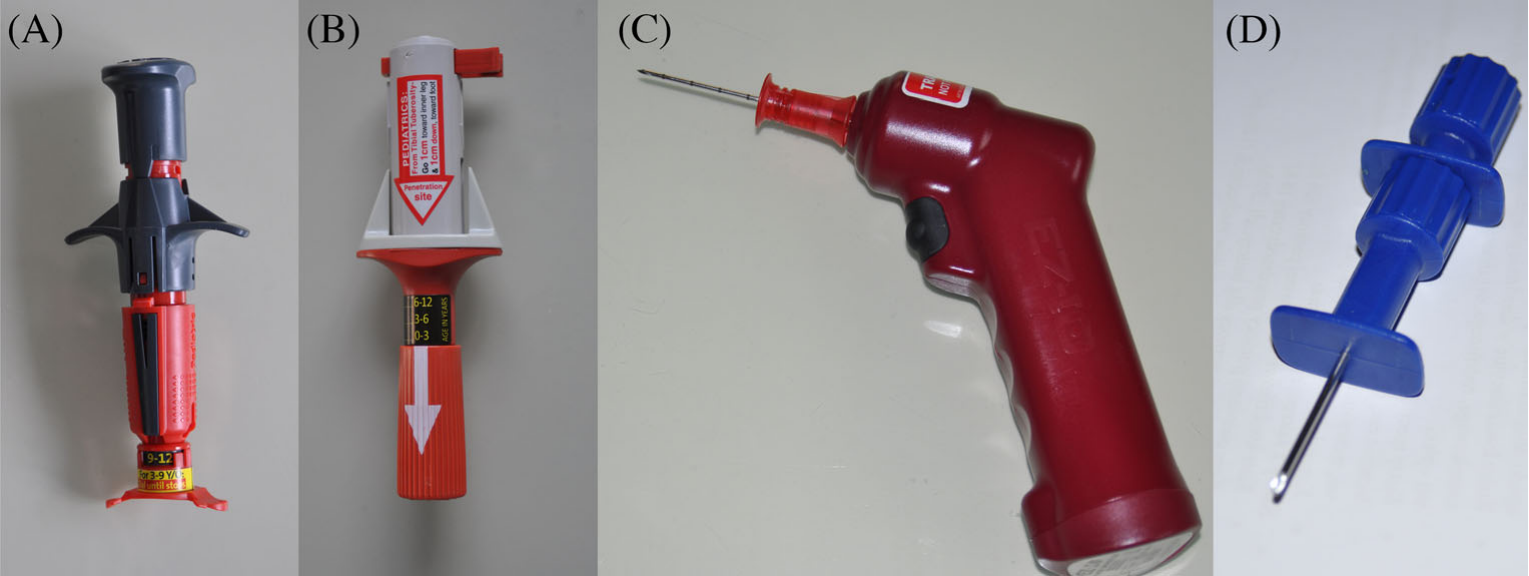
Intraosseous access devices used for this study were **a** NIO Pediatric, **b** BIG Pediatric, **c** EZ-IO, and **d** Jamshidi needle

The devices used for the study were the following (Fig. 1):

- The NIO Pediatric (New Intraosseous PerSys Medical, Houston, TX, USA) is a spring-loaded intraosseous device designed especially for the pediatric population, from age 3 to 12. It weighs approx. 100 g and is a single use spring-loaded device with a twist-to-unlock handle and a trigger mechanism. This single-package device contains an 18-gauge needle and stylet as well as needle stabilizer. It consists of location arrows on the device to assist in finding the correct intraosseous tibial location in pediatrics.
- The BIG Pediatric (Bone Injection Gun PerSys Medical, Houston, TX, USA) is a spring-loaded intraosseous device and was the first automatic intraosseous device to come to market. It weighs approx. 83 g and is a single use spring-loaded device with a “Pull out” safety latch and a safety stopper mechanism. This single-package device contains an 18-gauge needle and stylet, with an adjustable insertion depth depending on anatomic site. It is indicated for children less than 12 years of age.
- The IO drill ARROW® EZ-IO® (EZ-IO; Teleflex Medical Research Triangle Park, NC, USA) is a device composed of a battery-powered vascular access driver with an integrated driller stylet-tipped 15-gauge needle. Bone marrow is accessed by drilling a hollow needle to a preset depth. This study utilized the 15-mm-long needle that is recommended for placement in the proximal tibia in 3–39 kg patients.
- Jamshidi intraosseous needle (Jamshidi, Baxter HealthCare Corporation, Deerfield, IL, USA), it is 15-gauge disposable none marrow aspiration/IO infusion needle. Jamshidi needle is a manually inserted access with the use of pressure and rotation. Entry into medullary space in indicated by loss of resistance.

### Study procedure

**Fig. 2:**
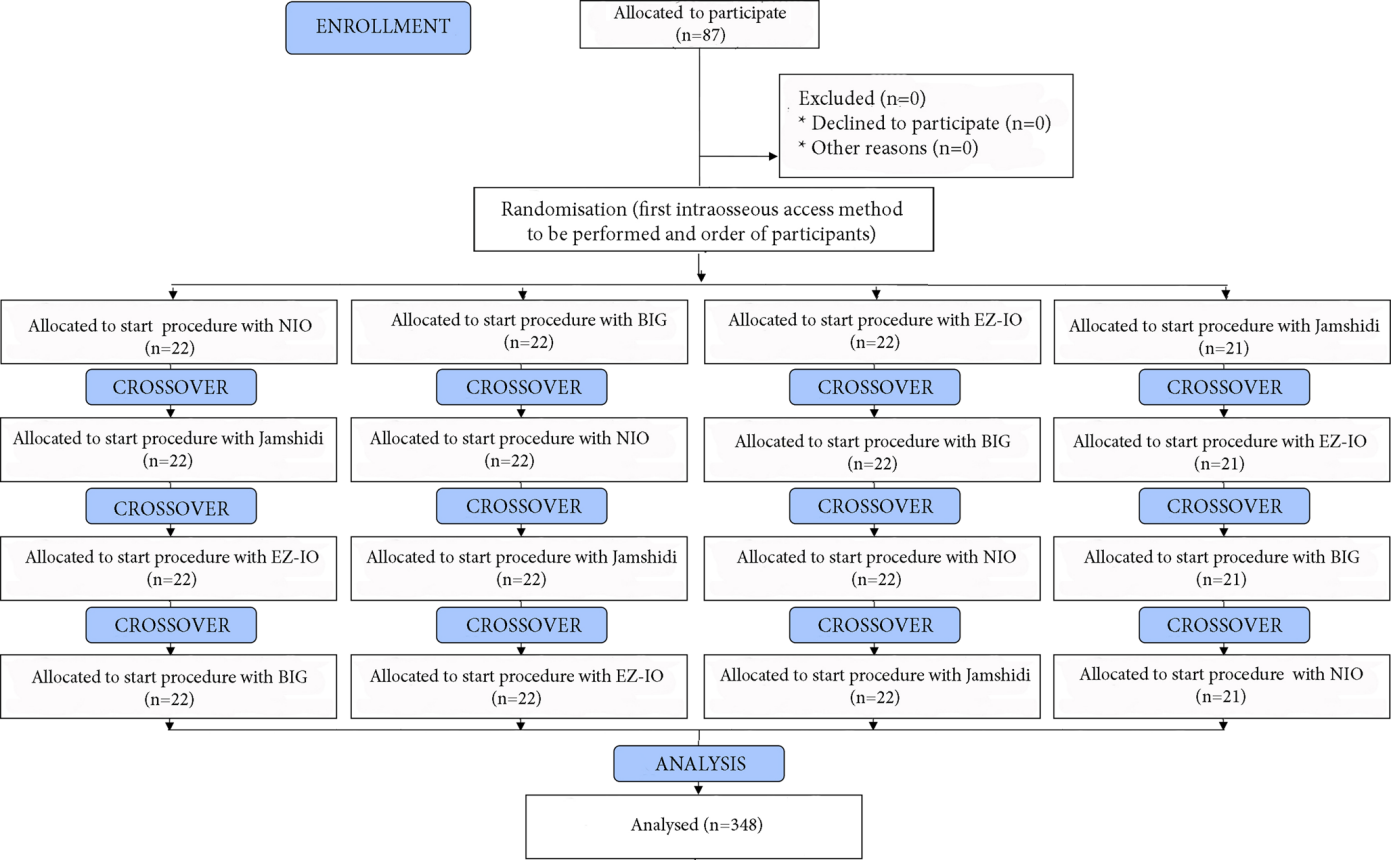
Flow chart of design and recruitment of paramedics according to CONSORT statement

Before the study, all paramedics participated in a 30-min lecture, covering the relevant basics of achieving an intraosseous access in pediatrics. At the end of the training, the instructor demonstrated the correct procedure of obtaining intraosseous access with all devices used in our study. Practicing with the devices prior to the study evaluation was not allowed. The order of both paramedics and intraosseous devices were randomized using a research randomizer program (www.randomizer.org; Fig. 2). Each paramedic was asked to perform a single attempt of intraosseous insertion with each device using the proximal tibia of the manikin. Before each insertion attempt, the paramedics were reminded that the “patient” needs emergent intraosseous access, in order to simulate a critical emergency situation. All insertion attempts were performed during ongoing chest compressions and a new needle was used for each insertion attempt. The same manikin tibia was used for the entire study, but the artificial skin was preplaced after each insertion attempt.

To minimize bias and to increase difficulty, the study investigators did not intervene with the procedure or provide any consultation or recommendation, and paramedics were not allowed to watch others perform the procedure.

### Outcome measures

The main outcome was the success rate of intraosseous cannulation on the first attempt, which was assessed by an independent. The secondary outcome was the time of intraosseous cannulation. We analyzed three time parameters:

- T1—time interval between grasping the intraosseous device out of the original packaging until completion of intraosseous needle placement
- T2—time since grasping the intraosseous device by the paramedic until stabilization of the injection site.
- T3—time since grasping the intraosseous device until connection to the infusion line.

After the procedure completion, each paramedic filled a questionnaire in which they subjectively rated the ease of intraosseous device use (1–10; 1 = very easy, 10 = very difficult), the ease of as well as the willingness to use the device in future CPR scenario.

### Power analysis

Power analysis was performed, which revealed that a sample size of 40 per group would provide 80% power to detect a moderate effect size difference of 1.0 (or approximately 1.0) standard deviation (SD) between the means at the alpha level of 0.05 (Statistica Software, version 12.5; StatSoft, Inc., Tulsa, OK, USA).

### Statistical analysis

All study data were entered into an electronic database (Microsoft Excel 2010; Poland) (Microsoft Corp, Redmond, WA, USA) and evaluated with the use of Statistica Package Software, version 13.1 (StatSoft, Tulusa, OK, USA). The value of *p*  <  0.05 was considered statistically significant.

The results were presented as absolute values (percentages), medians (interquartile ranges; IQRs), or means (±SD). The Kolmogorov–Smirnov test was applied to check for normal distribution. As this was a randomized crossover trial, pairing was taken into account in the statistical analysis. McNemar test was used for comparing the cannulation success rates. The two-sided Wilcoxon signed-rank test allowed to compare the procedure time. The paramedics’ subjective opinions were compared with the use of the Stuart–Maxwell test.

## Results

Eighty-seven paramedics (23 female, 26%) participated in this study and no paramedic had any previous experience with any intraosseous device. Fifty-one paramedics (13 female, 25%) worked in the EMS, while 36 paramedics (10 female, 28%) worked within hospital emergency departments (EDs). The mean age was 24 (IQR, 23–25) years, and the mean work experience was 0.5 (IQR, 0–1) years.

The correct location of intraosseous access was indicated in the vast majority of all insertion attempts (NIO 100%, BIG 90%, EZ-IO 90%, and Jamshidi 90%).

When using BIG, 7 insertion attempts were unsuccessful and were associated with incorrect applying of the device to the surface of limbs and tearing puncture while trying to introduce the needle, which resulted in the launch of the needle at the wrong angle. If using the EZ-IO, 5 paramedics incorrectly screwed the needle using an incorrect angle (instead of 90 degrees). Incorrect placements of Jamshidi needle were observed in 23 insertion attempts.

**Fig. 3:**
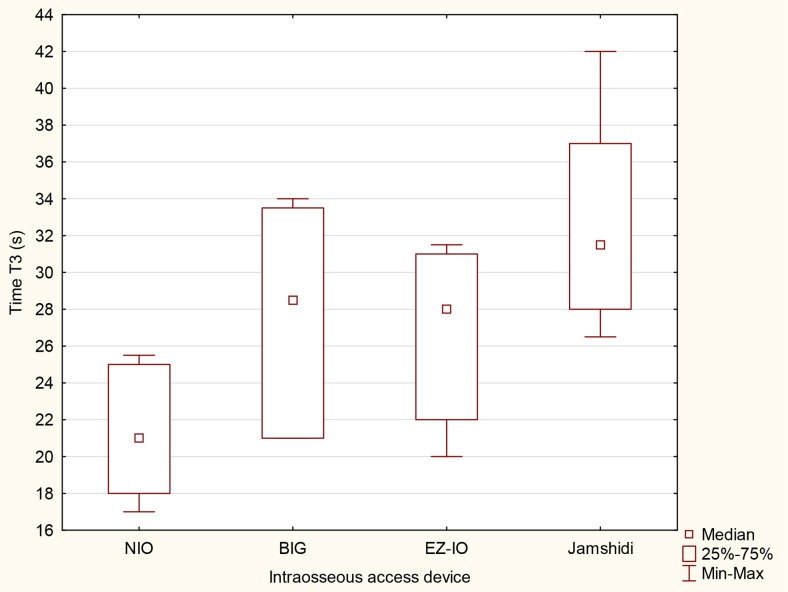
The median time since grasping IO device until the connection of the infusion line

The time required to obtain intraosseous access (time T1) varied and amounted to be 9 s [IQR, 8–12] for NIO, 12 s [IQR, 9–16] for BIG, 13.5 s [IQR, 11–17] for the EZ-IO, and 15 s [IQR, 13–19] for Jamshidi (Table 1). Time T2 (time to stabilize intraosseous access) are reported in Table 1 and T3 are presented in Fig. 3. A statistically significant difference was noticed between NIO and BIG (*p* = .011), NIO and EZ-IO (*p* = .013), NIO and Jamshidi (*p* < .001), as well as between BIG and Jamshidi (*p* = .035) and between EZ-IO and Jamshidi (*p* = .031).

**Table 1:** The test parameters. The results are presented as the median (interquartile ranges IQR)

Parameter	NIO	BIG	EZ-IO	Jamshidi	*p* value
T1—time to obtain the intraosseous access	9 (8–12)	12 (9–16)	13.5 (11–17)	15 (13–19)	NIO vs. BIG = .022 NIO vs. EZ-IO = .019 NIO vs. Jamshidi <.001 BIG vs. Jamshidi = .026 EZ-IO vs. Jamshidi = .031
T2—time to stabilize the intraosseous access	9 (8–12)	15.5 (12–19.5)	13.5 (11–17)	19 (16–23)	NIO vs. BIG = .002 NIO vs. EZ-IO = .007 NIO vs. Jamshidi <.001 BIG vs. Jamshidi = .017 EZ-IO vs. Jamshidi = .012
T3—time to connect the infusion line	21 (18–25)	28.5 (21–33.5)	28 (22–31)	31.5 (28–37)	NIO vs. BIG = .011 NIO vs. EZ-IO = .013 NIO vs. Jamshidi <.001 BIG vs. Jamshidi = .035 EZ-IO vs. Jamshidi = .033
The ease of the procedure	1.5 (0.5–1.5)	2.5 (2–4)	2 (2–3.5)	6 (5–7.5)	NIO vs. BIG <.001 NIO vs. EZ-IO <.001 NIO vs. Jamshidi <.001 BIG vs. Jamshidi <.001 EZ-IO vs. Jamshidi <.001

The paramedics subjectively evaluated each device after finishing the insertion attempt. The intraosseous device which proved the easiest to use was NIO with a median rating of 1.5 (IQR, 0.5–1.5) (Table 1). Of the paramedics, 91% would prefer to use NIO, 7%—EZ-IO, and 2% would use BIG in a real pediatric CPR setting.

## Discussion

We compared success rates, procedure times, and ease of procedure among four pediatric intraosseous devices during simulated pediatric CPR. The NIO-P has 100% first attempt success rate and is associated with statistically significantly improvement in (1) time to obtain intraosseous access; (2) time to stabilize the intraosseous access; and (3) time to connect the infusion line, compared to the other three intraosseous devices tested in our study (BIG, EZ-IO, and Jamshidi). The NIO-P seemed to be the easiest to use and was the overwhelmingly preferred device among paramedics. Performance of BIG and EZ-IO were similar and outperformed Jamshidi. We note that the difference in total time of procedure (T1 + T2 + T3) between NIO-P and other devices is small (fraction of a minute) and whether this difference will translate to any improvement in clinical outcomes is questionable. However, it appears the NIO-P is at least comparable in performance to the other intraosseous devices.

Rapid and high first attempt success rates in achieving a venous access are vital in a wide range of emergency settings including CPR. Failure rates for peripheral IV and central venous catheter in emergency situations can be as high as 40% [22]. Average time for peripheral IV is 2 to 26.7 min and that for central line placement is 8 to 11 min [22]. Multiple previous studies demonstrated that intraosseous access outperformed peripheral IV and central line placement on first attempt success rate and time of procedure in emergency situations [15, 16, 21]. In a study by Andersen et al., the authors demonstrated that any minute of delay in administration of epinephrine in pediatric cardiac arrest was associated with decreased chance of survival, return of spontaneous circulation and less favorable neurological outcomes [1]. With more than 5000 pediatric out-of-hospital cardiac arrest in the USA annually, establishing venous access is a critical component of a quality CPR [25]. Therefore, intraosseous access has potential to make a substantial impact on clinical outcomes.

The performance of NIO-P in our pediatric model is comparable to several published studies. In our study, first attempt success rate of NIO-P is 100%. In a simulated adult CPR simulation using human cadavers by our study group, the success rate of first attempt with NIO is 97% [22]. In another study by our group, the success rate of NIO in the adult cadaver tibia is 89% [22]. First attempt success is important because any first attempt failure means an extra 30–60 s will be spent on a second attempt on intraosseous access, which can potentially delay medical treatment and affect patient outcomes. Multiple punctures can potentially increase risk of complications, such as extravasation and infections. Our total procedure time for NIO-P placement averaged 39 s, which was significant longer as previously reported to be 17 s [24]. A plausible explanation is that our procedure time includes *time to connect the infusion line* (T3) and not mere achieving intraosseous access. The reason we measure T3 was that we believe connection of infusion line to the intraosseous device is also clinically important as starting infusion of drugs is the ultimate goal during CPR. If we only measure time to achieve access and stabilization, NIO-P required on average 18 s, almost equivalent to previous reports [22, 24]. Moreover, NIO-P was rated favorably on ease-of-use and an overwhelming majority (90.8%) preferred this device in a real-life scenario.

The BIG and EZ-IO showed similar performance in our study. The total median time of BIG and EZ-IO were 56 and 57 s, respectively. Both scored very closely on ease-of-use. While 7% preferred EZ-IO in a real-life scenario, only 2% would use BIG. This is similar to previous report by Shavit et al. that showed EZ-IO was the preferred device over BIG in a turkey bone model [18]. One plausible for our finding is that the EZ-IO required fewer steps versus the BIG. Compared to the BIG, which requires a number of steps releasing the needle (non-dominant hand holds the device perpendicular to the skin, pull out safety pin and the dominant hand release the spring mechanism), the EZ-IO has the theoretical advantage of being more intuitive and easier to use with a single step (one simultaneous motion of squeezing trigger and the needle is effortlessly drilled into the bone). An additional advantage of the EZ-IO is that its needles are color-coded based on weight for ease-of-use. The BIG required the user to twist the barrel to adjust the needle depth based on patients’ age, which might not be available in real-life scenarios. Although user preferences do not affect time of access, it reflects that more intuitive handling of EZ-IO and could have positive implications in clinical practice. Jamshidi, a manual IO device, had the lowest success rate, required longest time to perform and had the lowest preference rate. Our finding is supported by multiple prior studies that drill-assisted IO devices were had higher success rates and more reliable than manual devices [5, 9]. Jamshidi needle required manual force to drive the needle forward. Too little force may not penetrate cortical bone, especially in older children, while excessive force may cause needle to bend.

Although rare, accidental disconnection of catheters has been described during CPR and during interhospital transfer [8, 17]. A dislodged needle may prevent medications reaching central circulation and in cases where large volumes of fluids are infiltrated may lead to compartment syndrome [20]. A Scandinavian survey 1800 cases in intraosseous placement, there was a 12% incidence of needle displacement and extravasation [6]. The BIG device requires the user to attach a “safety latch” to the intraosseous needle for stabilization; however, this extra step potentially causes needle dislodgement. The NIO-P potentially minimizes this risk with a built-in stabilizing manifold in the needle hub that secures the intraosseous needle during placement.

There are several limitations in our study. First, we conducted our study using a manikin model which might limit our external validity in real patients. However, the Laerdal Manikin have been widely used in CPR research, including intraosseous device studies [9, 19]. Manikins do not have medullary cavity therefore did not allow us to confirm “successful” with aspiration of marrow or injection of fluid. However, we used independent instructors who were experienced in advanced life support and anatomy to confirm a successful intraosseous placement. Our findings can only be applied to a 6-year old pediatric patient as dictated by our manikin. Older children, with a thicker bony cortex, may need more force to access the medullary cavity, thus yield better results with power-assisted devices. We also could not assess the maximum rate of fluid infusion and complications associated with these devices.

## Conclusions

In summary, our study found that NIO-P is superior to BIG, EZ-IO, and Jamshidi. NIO-P achieved the highest first attempt success rate. We found a small but statistically significant difference between insertion time of NIO-P and other devices and easiest to operate even by non-experts. Further studies in cadavers and human subjects are needed in order to confirm our findings.

## Abbreviations

AHA: American Heart Association
CPR: Cardiopulmonary resuscitation
ERC: European Resuscitation Council
EMS: Emergency Medical Service
FDA: Food and Drug Administration
IO: Intraosseous access
IQR: Interquartile range
IV: Intravenous access
SD: Standard deviation
